# Access to general practitioner services amongst underserved Australians: a microsimulation study

**DOI:** 10.1186/1478-4491-10-1

**Published:** 2012-01-22

**Authors:** Deborah J Schofield, Rupendra N Shrestha, Emily J Callander

**Affiliations:** 1 NHMRC Clinical Trials Centre, University of Sydney, Medical Foundation Building, 92-94 Parramatta Road, Camperdown, NSW 1450, Australia

## Abstract

**Background:**

One group often identified as having low socioeconomic status, those living in remote or rural areas, are often recognised as bearing an unequal burden of illness in society. This paper aims to examine equity of utilisation of general practitioner services in Australia.

**Methods:**

Using the 2005 National Health Survey undertaken by the Australian Bureau of Statistics, a microsimulation model was developed to determine the distribution of GP services that would occur if all Australians had equal utilisation of health services relative to need.

**Results:**

It was estimated that those who are unemployed would experience a 19% increase in GP services. Persons residing in regional areas would receive about 5.7 million additional GP visits per year if they had the same access to care as Australians residing in major cities. This would be a 18% increase. There would be a 20% increase for inner regional residents and a 14% increase for residents of more remote regional areas. Overall there would be a 5% increase in GP visits nationally if those in regional areas had the same access to care as those in major cities.

**Conclusion:**

Parity is an insufficient goal and disadvantaged persons and underserved areas require greater access to health services than the well served metropolitan areas due to their greater poverty and poorer health status. Currently underserved Australians suffer a double disadvantage: poorer health and poorer access to health services.

## Background

The burden of disease is not equally distributed amongst Australians. Those with low socioeconomic status generally have a lower life expectancy, and are more likely to suffer from chronic disease and have health risk factors [1-3]. Those with the lowest socioeconomic status are reported to have around one-third greater burden of disease than those with the highest socioeconomic status [4].

The socioeconomic differences in mortality within Australia have been documented, with it being concluded that if death rates in the lowest socioeconomic areas could be reduced to that of the least disadvantaged areas, all-cause mortality could be reduced by up to 70% [5]. Successive reports conducted by the Australia Institute of Health and Welfare show that people from low socioeconomic backgrounds have more risk factors for chronic disease and greater prevalence of certain diseases including diabetes, asthma, diseases of the circulatory system, mental problems, and arthritis [1-3,6].

One group often identified as having low socioeconomic status, those living in remote or rural areas, are often recognised as bearing an unequal burden of illness in society. Those living in rural and remote areas experience poorer health status, lower life expectancy and are more susceptible to illness and injury [7].

A commonly recognised problem within the health system is the maldistribution of the health workforce, with rural areas having less access to general practitioner (GP) services, even though, on a range of measures, rural populations experience poorer health than those in capital cities [8]. It has been reported that in 2008, there were 376 full-time equivalent (FTE) medical practitioners per 100 000 individuals in major cities, with this figure falling to 187 FTE medical practitioners per 100 000 individuals in very remote areas [9].

While these studies indicate that the workforce is not equitably distributed, what is not known is the extent of unmet demand for GP services. This demand is more than a per capita comparison, but is driven by health and socioeconomic status as well as demographic factors. Some univariate comparison of GP visits [10] inadvertently mask the inequality of the distribution of GP services relative to need because the results are not adjusted to take account of factors such as the poorer health status of the economically disadvantaged [11], or the relatively higher proportion of elderly people in the low income groups [12].

This paper includes an assessment of the equity of the distribution of GP services in Australia, looking at how much lower the utilisation of GP services in rural areas is compared to those in major cities. It also provides a simulation to demonstrate how many additional GP services underserved rural areas (underserved by comparison with major cities) would benefit from if there was universal utilisation of GP services to all Australians at the current level of persons residing in major cities.

## Methods

The rationale for 'modelling demand for medical services' (MedDemandMOD) was to develop a model of demand for GP services that captured more than the typical age/sex utilisation of services. Rather, it was designed so that it could capture the impact of demographic determinants such as age, sex and regional factors such as remoteness, and socioeconomic factors such as personal income and employment status and indicators of need such as health status.

The 2005 National Health Survey (NHS) undertaken by the Australian Bureau of Statistics (ABS) is used as the data source, which collected information about the health of Australians in 2004-05. The NHS has the advantage of being a nationally representative sample survey with a range of socio-economic and health determinants of medical demand.

The use of GP services in the two weeks preceding the survey was recorded for the population aged 15 and over. The frequency of GP visitations was recorded by the ABS to be not at all, once, twice or three times or more in the previous two weeks. This categorical information has been converted into a continuous variable of zero, one, two, and three GP visits. While collapsing information into the three visits category is not ideal as there is a loss of information, this is a constraint of the data set used. However, it is not felt that this will significantly affect the accuracy of predicted GP visits as only less than 1% of the population stated they visited a GP three times or more in the previous two weeks. By setting this category at three visits per fortnight, this will have produced a slightly conservative estimate.

Other variables on the NHS that were utilised in this study were income, geographic location, employment status and health status. The gross weekly equivalised cash income was recorded on the NHS, with responses grouped into deciles by the ABS. This was then grouped into income terciles for this study to ensure adequate record numbers in each group. Cut-off points for the income terciles were: tercile 1 (up to $345); tercile 2 ($346-$654); tercile 3 ($655 and over). In 2005, the average full time adult ordinary time earnings were $1022 per week (this did not include part time income or income for social security payments) [13], and in 2006, the median personal income from all sources was $400 to $499 per week [14]. Geographic location was recorded on the NHS into three groups - major cities, inner regional areas and other areas. Employment status was grouped from the original NHS data into three groups - those employed full time or part time; those unemployed and looking for full time or part time work; and those not in the labour force. Health status was recorded on the NHS as excellent, very good, good, fair or poor from an individual's assessment of their general health. This was then grouped for this study into three groups - excellent and very good; good; fair and poor.

One of the first challenges was to account for unmet demand. Although an imperfect measure, it was assumed that in capital cities, where there is the highest concentration of GPs, demand was fully met. The limitation is that any unmet demand in capital cities is not captured. The total demand in underserved areas was then estimated to be the services that would be consumed if persons from other areas had the same supply of medical services as persons in capital cities given their health status (which is poorer), their demographic characteristics and factors that might constrain their use, such as personal income (to meet out of pocket costs).

MedDemandMOD was used to simulate what would be the total demand for GP services in remote and regional areas, had the utilisation of GP services in these areas been the same as in the major cities. This was done using SAS V9.2 software (SAS Institute Inc., Cary, NC, USA). A multidimensional matrix of the average use of GP services, with the dimensions being the socio-demographic and health status variables listed in Table 1, was set up. The total number of matrix cells was 648. A multinomial logistic regression model was used to analyse the association of the matrix variables with the use of GP services, and to test if they were significant determinants of service use. No visit to a GP (i.e. "zero" number of visit) was chosen as a reference group in the model. The goodness of fit for the model was checked. The lack of fit tests of the model using the deviance and Pearson goodness of fit tests did not provide any evidence of lack of fit of the model to the data.

**Table 1:** Variables used to index the multidimensional array of average GP services used in the previous 2 weeks

Variable	Categories	Number of categories
Sex	Male, Female	2
Age group	15-20, 21-44, 45-64, 65 + years	4
Personal income tercile	tercile 1, tercile 2, tercile 3	3
Remoteness	Major cities of Australia, Inner regional Australia, Other more remote areas	3
Self reported health status	Excellent/very good, good, fair/poor	3
Employment Status	Employed, Unemployed (looking for work), Not in the labour force	3

Using the simulation model, a deterministic model, we estimated the same number of services that would be used by the rural population if they were to use the same number of services as those in the major cities with the same characteristics (age, sex, employment status, health status and income).

## Results

Table 2 shows the results of the multinomial logistic regression model for the use of GP services in the two weeks prior to the survey. The regression analysis shows that age, sex, employment status, remoteness and self reported health status were associated with the number of times a GP was visited in the two weeks prior to the survey. People in the 65 or more years age group were significantly more likely to visit a GP one or two times per fortnight than the younger population. Males were significantly less likely than females to visit a GP. The likelihood of consulting a GP one time compared to none in the two weeks prior to the survey was significantly lower for people in regional communities than people in major cities. However, the analysis did not provide any evidence of the association between where they live and their likelihood of consulting a GP two or three times or more compared to none in the two weeks prior to the survey. People with fair and poor health status were significantly more likely to visit a GP compared to people with excellent to good health status.

**Table 2:** Association between the number of GP visits in the last two weeks and socio-demographic variables, relative risk ratios and their 95% CIs.

Variables	Number of times GP consulted per fortnight			Overall p-value
		
	One visit*	Two visits**	Three or more visits***	
*Age group*				< 0.0001
15 - 19	**0.39 (0.30, 0.49)**	**0.43 (0.26, 0.71)**	0.60 (0.24, 1.52)	
20 - 44	**0.53 (0.46, 0.61)**	**0.55 (0.41, 0.75)**	0.68 (0.40, 1.16)	
45 - 64	**0.63 (0.55, 0.73)**	**0.67 (0.50, 0.89)**	0.68 (0.40, 1.17)	
65 +	Reference	Reference	Reference	
*Sex*				< 0.0001
Male	**0.72 (0.66, 0.79**)	**0.70 (0.57, 0.87)**	**0.58 (0.38, 0.89)**	
*Employment status*				0.0064
Employed	**0.78 (0.68, 0.89)**	0.78 (0.58, 1.05)	0.96 (0.57, 1.63)	
Unemployed (looking for work)	0.99 (0.74, 1.32)	1.17 (0.63, 2.18)	0.63 (0.19, 2.12)	
Not in the labour force	Reference	Reference	Reference	
*Remoteness*				< 0.0001
Inner regional	**0.76 (0.68, 0.86)**	0.84 (0.65, 1.08)	0.79 (0.50, 1.24)	
Other regional	**0.80 (0.70, 0.91)**	0.96 (0.72, 1.27)	0.76 (0.43, 1.33)	
Major cities	Reference	Reference	Reference	
*Personal income*				0.5803
First tercile	1.12 (0.96, 1.30)	1.10 (0.78, 1.55)	1.14 (0.60, 2.16)	
Second tercile	1.10 (0.97, 1.25)	1.16 (0.86, 1.56)	0.85 (0.49, 1.47)	
Third tercile	Reference	Reference	Reference	
*Health status*				< 0.0001
Exec/V Good	**0.37 (0.33, 0.42)**	**0.20 (0.16, 0.26)**	**0.10 (0.06, 0.17)**	
Good	**0.55 (0.48, 0.62)**	**0.28 (0.21, 0.36)**	**0.34 (0.22, 0.51)**	
Fair/Poor	Reference	Reference	Reference	

Bold relative risk ratios are statistically significant at 5% significance level.

*Relative risk ratios of consulting GP one time compared to none

**Relative risk ratios of consulting GP two times compared to none

***Relative risk ratios of consulting GP three or more times compared to none

Those who live in regional areas have fewer GP visits per year, on average, than those in major cities. As expected, those with poor health have a greater number of average GP visits per year than those with better health. Those from regional areas are, generally speaking, from lower income declines than those in major cities (Table 3).

**Table 3:** Annual GP services if access universally the same as in major Australian cities, 2004-05^1^

Simulation variables	Base	Simulation	Difference		Base	Simulation	Difference	Difference
	**No**.	**No**.	**No**.	%	Mean	Mean	**No**.	%
**Employment Status**								

Employed	51,332,000	54,197,000	2,865,000	6	5.1	5.4	0.3	6
Unemployed (looking for work)	3,054,000	3,626,000	573,000	19	6.6	7.9	1.2	19
Not in labour force	51,846,000	54,061,000	2,214,000	4	9.9	10.3	0.4	4
**Total**	106,232,00	111,884,000	5,652,000	5	6.7	7.1	0.4	5
**Tercile of personal income**								

First tercile (lowest)	36,867,000	38,604,000	1,736,000	5	9.1	9.5	0.4	5
Second tercile	42,437,000	45,221,000	2,784,000	7	6.8	7.3	0.5	7
Third tercile	26,928,000	28,060,000	1,132,000	4	4.9	5.1	0.2	4
**Total**	106,232,000	111,884,000	5,652,000	5	6.7	7.1	0.4	5
**Health status**								

Excellent-very good	40,365,000	43,049,000	2,684,000	7	4.6	4.9	0.3	7
Good	30,887,000	32,380,000	1,493, 000	5	7.1	7.4	0.3	5
Fair-Poor	34,980,000	36,455,000	1,475,000	4	13.9	14.5	0.6	4
**Total**	106,232,000	111,884,000	5,652,000	5	6.7	7.1	0.4	5
**Remoteness**								

Major city	74,245,000	74,245,000	0	0	7.0	7.0	0	0
Inner regional	19,371,000	23,196,000	3,825,000	20	6.2	7.4	1.2	20
Other regional	12,616,000	14,442,000	1,826, 000	14	6.6	7.5	1.0	14
**Total**	106,232,000	111,884,000	5,652,000	**5**	6.7	7.1	0.4	5

^1^Presented to the nearest 1000

MedDemandMOD was then used to simulate how many more services people in disadvantaged groups would use if they had the same access as Australians in major cities given their lower income, poorer health status and so on.

Based on the simulation, Australians in regional areas would benefit from about 5.7 million additional GP visits per year (Table 3); about 3.8 million (a 20% increase) for inner regional residents and about 1.8 million for residents of other regional areas (a 14% increase). The average number of visits would rise from 6.2 to 7.4 per annum in inner regional areas and from 6.6 to 7.5 in other regional areas. Their use would then be higher than for persons living in major cities (who averaged 7.0 GP visits per annum), reflecting both the current lack of services and poorer health of rural Australians.

The simulated percentage increase in use of GP service utilisation associated with those in the lowest tercile of income was lower than for those in the second tercile (table 3). This was due to the large proportion of aged individuals being in the lowest tercile of income, and the model revealed greatest gains for younger age groups, rather than older age groups (i.e. there was smaller difference in the utilisation of GP services in rural areas and major cities for older individuals than for younger individuals).

When averaged across all regions, Australians who were unemployed (looking for work) would experience a 19% increase in GP visits. Those in the lowest personal income tercile would experience a 5% increase in GP visits, and those in the second tercile would experience a 7% increase in GP visits.

Nation-wide there would be an additional 5%, or 5.7 million GP visits, in 2004-05 if those in regional areas had the same access to GP services that those in major cities do.

## Discussion

There is ample evidence that ill health is associated with both poverty and rurality [4,8]. Accordingly, those from rural areas will have a higher need for GP services. However, the results of this study has shown that in 2004-05 those in regional areas visited a GP fewer times, on average, than those in major cities. This indicates that there may be a shortfall in demand and those in regional areas do not have the utilisation of GP services they require. Indeed, it is shown that if those in regional areas had the same utilisation of GP services as those in major cities, they would actually have a higher number of average GP visits per year. It is estimated that there is a shortfall in demand of around 5.7 million GP visits annually for those in regional areas.

This paper does have limitations which must be noted. Firstly, it is assumed that demand is fully met in major cities. While GPs in major cities have traditionally worked shorter hours than their counterparts in rural areas in Australia (indicating that they may be less likely to be overworked), there is less of a difference in younger cohorts, with younger GPs in rural areas working only marginally more hours than their major city counterparts [15]. Within Australia there is debate over what is considered an 'adequate' number of GP visits. For example, the Australian Medical Workforce Advisory Committee has used the number of services in major regional areas as a benchmark figure; however this has been disputed on the basis that there was not good evidence that people who live in major cities received more health care visits than would be ideal [16]. If demand is not fully met in major cities, then the figures presented in this paper underestimate the shortfall in utilisation experienced by those in regional areas, as currently those in major cities have the best access to GP services. Secondly, the inequality associated with aboriginality has not been assessed. This is largely a data limitation, as the 2005 NHS does not identify responders who have Torres Strait Islander or Aboriginal heritage. This is a limitation as it is well documented that Indigenous Australians have a far lower health status and life expectancy - 11.5 years for males, and 9.7 years for females, than their non-Indigenous counterparts, and mainly reside outside major cities (only 31% of the Indigenous population live within major cities) [17,18], and thus have a particularly high need for access to GP services. Thirdly, as indicated in the methods sections some variables, such as income, health and number of GP services had to be collapsed into fewer categories to allow a sufficient number of records in each cell. Thus, it was not possible to estimate service utilisation specifically for smaller subgroups such as those living in very remote areas. Finally, it is assumed that if additional services are provided in rural areas, these will be taken up by the rural population. However, it should be noted that there are other barriers to the utilisation of health care services, such as cultural constraints, trust in health care systems, and availability of transport to services [19-21].

Those in regional and rural areas have far poorer health status than their urban counterparts [7]. In spite of this they currently do not have the same utilisation of GP services as those in major cities. Thus, there is a large shortfall in meeting the health care needs of those in rural areas - geographically the most ill group of the Australian population. This confirms the view raised in 1971 by Julian Tudor Hart that those with poorer health receive less access to medical care than those with better health [22]. While the costs of health care have been noted to be a barrier for 6% of the Australian population in seeking GP services, previous studies have found no difference between cost being a barrier to GP service vists between those in rural areas and major cities [23].

The *National Reform Agenda *calls for a health care system that is effective and efficient and focuses on preventing chronic disease in order to improve the health of Australians [24]. However, the current distribution of health care services will not allow for this goal to be met, as those from low socioeconomic groups and regional areas - who are the most ill in society, do not have adequate utilisation of health care services to meet their need. Continuing to not meet the needs of those who are most in need will hinder the overall improvement of the health of the Australian population, and may increase the inequalities between those with a high socioeconomic status and those with low socioeconomic status [25-27].

Current workforce planning focuses on the number of GPs per capita or per age/sex population [28-30]. As a result, workforce plans strive to increase GP numbers to provide the same number of GPs to all Australians according to simple demographic parameters which do not reflect demand or need for GP services. Those in regional areas have a greater need or demand for access to GP services than those in major cities.

Australian models of 'demand' have tended to be treated simply as the services currently supplied. The distribution of services by age and sex has then been used as the basis for projecting future 'demand' [10-13]. The limitation of this approach is that existing unmet demand is not defined, and current inequalities resulting from the misdistribution of the health workforce are implicitly assumed to continue. Sometimes, in the absence of estimates of demand, workforce requirements are based on other proxy measures such as a study of the radiology workforce which relied on vacancy rates, radiologist to population ratio, and reports of work over-load as indicators [12].

There is a real need to pursue policies that deliver affordable services to disadvantaged areas. Currently polices which promote service delivery of this type include establishing rural clinical schools and schools in less well served areas such as in western Sydney to train doctors within underserved areas. This has the advantage of attracting students who come from disadvantaged areas, who know and understand the area in which they are likely to work [31]. Bonded medical places have been another approach which ensures that new medical places go to students who agree to spend at least part of their career practicing in an underserved area [32]. Incentives to increase bulk billing are also important to ensure affordable access to services provided in rural areas in particular [33]. Internationally, motives to attract health professionals to low socio-economic areas have included incentive salaries for health professionals who practice in disadvantaged areas [34]. Another option for very small and remote communities where it is difficult to recruit GPs, is models of funding which allow the 'cashing out' of Medicare subsidies to cover a viable health service model that may feed other types of multidisciplinary and multipurpose services.

## Conclusions

In this study we have demonstrated that having the same per capita utilization of GP services between rural and non-rural areas is not a sufficient goal for government and that disadvantaged persons and underserved areas require greater access to health services than the well served metropolitan areas due to their greater poverty and poorer health status.

## Competing interests

The authors declare that they have no competing interests.

## Authors' contributions

DS conceived of the study, led the development of the microsimulation model and participated in the drafting and editing of the manuscript, data analysis and interpretation of the findings. RS participated in the drafting and editing of the manuscript, statistical data analysis and interpretation of the findings. EC participated in developing the microsimulation modeling, the drafting and editing of the manuscript, and interpretation of the findings. All authors read and approved the final manuscript.
